# Diagnostic utility of allergy tests to predict baked egg and lightly cooked egg allergies compared to double‐blind placebo‐controlled food challenges

**DOI:** 10.1111/all.15797

**Published:** 2023-07-07

**Authors:** Marta Krawiec, Suzana Radulovic, Ru‐Xin Foong, Andreina Marques‐Mejias, Irene Bartha, Matthew Kwok, Zainab Jama, Faye Harrison, Cristian Ricci, Gideon Lack, George Du Toit, Alexandra F. Santos

**Affiliations:** ^1^ Department of Women and Children's Health (Pediatric Allergy), School of Life Course Sciences, Faculty of Life Sciences and Medicine King's College London London UK; ^2^ Children's Allergy Service Evelina London Children's Hospital, Guy's and St Thomas' Hospital London UK; ^3^ Peter Gorer Department of Immunobiology, School of Immunology and Microbial Sciences King's College London London UK; ^4^ Africa Unit for Transdisciplinary Health Research (AUTHeR) North‐West University Potchefstroom South Africa

**Keywords:** baked egg allergy, basophil activation test, diagnosis, egg allergy, food allergy, ovalbumin, skin prick test, specific IgE

## Abstract

**Background:**

Double‐blind placebo‐controlled food challenges (DBPCFC) are the gold‐standard to diagnose food allergy. However, they can cause allergic reactions of unpredictable severity. We assessed accuracy of current and new diagnostic tests compared to DBPCFC to baked egg (BE) and to lightly cooked egg (LCE).

**Methods:**

Children aged 6 months to 15 years were assessed for possible egg allergy as part of the BAT2 study (NCT03309488). They underwent clinical assessment, skin prick test (SPT), specific IgE (sIgE) and basophil activation test (BAT). The results of the tests were compared with DBPCFC outcomes to both BE and LCE.

**Results:**

A total of 150 children underwent DBPCFC to BE, 60 (40%) reacted to and 85 (57%) tolerated BE and 5 (3%) had inconclusive oral food challenges (OFC). Seventy‐seven children tolerant to BE had DBPCFC to LCE and 16 reacted. The test within each modality with the best diagnostic performance for BE allergy was as follows: SPT to egg white (EW) (AUC = 0.726), sIgE to EW (AUC = 0.776) and BAT to egg (AUC = 0.783). BAT (AUC = 0.867) was the best test in the younger than 2 years age group. Applying 100% sensitivity and 100% specificity cut‐offs, followed by OFC, resulted in 100% diagnostic accuracy. BAT enabled the greatest reduction in OFC (41%). Using sIgE followed by BAT allowed to reduce the number of BATs performed by about 30% without significantly increasing the number of OFC.

**Conclusions:**

The best diagnostic test was BAT to egg in terms of diagnostic accuracy and reduction in number of OFC. Using sIgE to EW followed by BAT required fewer BATs with sustained OFC reduction and diagnostic accuracy.

## Graphical abstract

All participants had DBPCFC to baked egg and, if this was negative, also DBPCFC to lightly cooked egg; SPT, sIgE and BAT were performed in parallel. The test with the best diagnostic performance was BAT, followed by sIgE and SPT. Applying 100% sensitivity and 100% specificity cut‐offs allowed 100% accuracy. Using tests sequentially reduced the number of OFCs (and BATs).Abbreviations: BAT, basophil activation test; DBPCFC, double‐blind placebo‐controlled food challenge; EW, egg white; NCT, national clinical trial; OVA, ovalbumin; OVM, ovomucoid; OFC, oral food challenge; OFC+, positive oral food challenge; SPT, skin prick test; sIgE, specific IgE

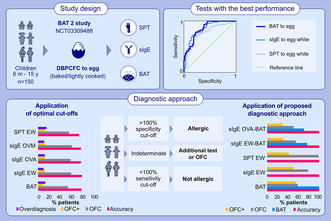

**Abbreviations**
BAT: basophil activation test
DBPCFC: double‐blind placebo‐controlled food challenge
EW: egg white
NCT: national clinical trial
OFC: oral food challenge
OFC+: positive oral food challenge
OVA: ovalbumin
OVM: ovomucoid
sIgE: specific IgE
SPT: skin prick test

## 1 INTRODUCTION

Egg allergy is one of the most common food allergies in childhood.^1^, ^2^ Most egg allergic children tolerate baked egg (BE) and resolve their egg allergy spontaneously over time.^3^, ^4^, ^5^ However, at the time of diagnosis, most children with suspected egg allergy are either avoiding BE strictly or eating small amounts, and this leaves uncertainty in terms of the best advice to give about consumption of BE, namely the amount likely to be tolerated and whether the introduction of BE needs to be done as an oral food challenge (OFC) in the hospital setting or can be done at home in the community.^6^

Previous studies have shown that allergy tests currently used in clinical practice, such as skin prick test (SPT) and specific IgE (sIgE) to allergen extracts, are not very precise predictors of the allergic status to BE, with sensitivity and specificity being 54/68% and 80/74% respectively, according to a recent meta‐analysis.^7^ Allergen components, namely egg major allergen ovomucoid (OVM), showed to be more informative than sIgE to egg extract to identify patients with persistent and BE allergy in some studies but not others.^8^, ^9^ Double‐blind placebo‐controlled food challenges (DBPCFC) remain the gold‐standard to diagnose BE allergy; however, they are resource‐intensive and can cause allergic reactions of unpredictable severity. Thus, improved tests are needed to enable an accurate diagnosis and reduce the number of referrals for OFC.

We aimed to assess accuracy of new diagnostic tests, namely the basophil activation test (BAT), as well as current tests, such as SPT and sIgE, compared to DBPCFC to BE and to lightly cooked egg (LCE). The BAT assesses the expression of activation marker CD63 on the surface of blood basophils by flow cytometry following stimulation with the allergen or controls.^10^ Our hypothesis was that the BAT, being a functional test, was more accurate than tests that measure the presence of allergen‐sIgE to confirm the diagnosis of BE and LCE allergies. This hypothesis follows on from our previous studies on peanut allergy,^10^, ^11^ where BAT showed to be a superior diagnostic test and to reflect the function of IgE in its ability to induce basophil activation following stimulation with the allergen and therefore to induce allergic symptoms.

## 2 METHODS

### 2.1 Study design

The BAT 2 Study (NCT03309488) was a cross‐sectional diagnostic study in which children with suspected IgE‐mediated allergy to either cow's milk, egg, sesame or cashew nut were prospectively recruited from specialised tertiary Paediatric Allergy clinics in London to undergo a diagnostic work‐up to confirm or refute the diagnosis. The present manuscript reports results for the egg study according to the STARD guidelines. Participants were referred by many different clinicians working in Paediatric Allergy specialist clinics at the Evelina London Children's Hospital or private clinics in London, and a screening telephone visit was undertaken by the study team to confirm eligibility. Children assessed for possible egg allergy were submitted to DBPCFC to BE and, if they passed this, to DBPCFC to LCE. Participants' parents or carers completed food frequency questionnaires (FFQ) and 7‐day food diaries, and children underwent clinical evaluation, SPT, sIgE testing to allergen extracts and to allergen components, BAT and OFC. All study participants had DBPCFC, except for infants younger than 12 months who had open OFC. After completion of the DBPCFC, participants' parents or carers filled in FFQ to egg every 2 months for 2 years.

### 2.2 Participants

Children aged 6 months or more and younger than 16 years at the time of consent were prospectively and sequentially recruited. Suspected IgE‐mediated egg allergy was defined by a history of an immediate‐type allergic reaction to any form of egg, or the presence of sIgE to egg as documented by SPT greater or equal to 1 mm and/or serum sIgE greater or equal to 0.10 KU/L. Infants and children with no history of regular consumption of an age‐appropriate amount of egg were included as their allergic status to egg remained unclear in the absence of uneventful egg consumption. Children tolerating small amounts of BE were advised to avoid BE for at least 2 days prior to blood collection for BAT and sIgE. Exclusion criteria are listed in the Supporting Information. The study was approved by the London – Westminster Research Ethics Committee (reference 17/LO/0296) and the UK Health Research Authority. Informed consent was obtained from parent or guardian and assent was obtained from the child before any study procedures.

### 2.3 Skin prick testing

SPT was performed using a single‐head metal lancet, a positive control (10 mg/mL histamine dihydrochloride), a negative control (50% glycerol and 50% buffered saline), egg white (EW) extract (ALK Abello) and fresh foods, including raw egg and BE (the latter using slurry made up of 1 g of the challenge food in 10 mL of saline). Skin weal diameter was recorded in milimeters after 15 min. The size of the wheal was determined as the arithmetic average of two perpendicular diameters including the longest one. The positive control test needed to be ≥3 mm, and the negative control test needed to be 0 mm. If the saline negative control test was ≥1 mm or the histamine positive control was ≤3 mm, SPT was considered inconclusive.

### 2.4 IgE and IgG4 testing

Venepuncture was performed prior to the OFC. Blood collection was repeated if the OFC was performed more than 6 months after blood collection. Total IgE, sIgE and IgG4 to egg, EW, ovalbumin and ovomucoid were determined using the standardised immunoenzymatic assay ImmunoCAP (Thermofisher). For IgE levels above 100 KU_A_/L, serial dilutions were performed to determine the exact serum IgE level.

### 2.5 Basophil activation test

Blood was collected in lithium heparin‐containing tubes, and the BAT was performed on the same day of blood collection. Per condition, 100 μL of whole blood was incubated with the same volume of egg extract (ALK‐Abello) or baked EW (Sigma‐Aldrich) diluted in RPMI (GIBCO). Baked EW was prepared as a single batch by heating in a oven at 180°C for 20 min and stored at −80°C thereafter. Anti‐IgE (1 μg/mL; Sigma‐Aldrich) and formyl‐methionyl‐leucylphenylalanine (fMLP, 1 μM; Sigma‐Aldrich) were used as positive controls and RPMI alone as a negative control. Cells were stained with CD123‐FITC, CD203c‐PE, HLA‐DR‐PerCP, and CD63‐APC (all Biolegend) which had been lyophilised in individual tubes as a single batch at the start of the study. Flow cytometry was performed using FACS Fortessa with FACSDiva software (BD Biosciences). The flow cytometry data were analysed using FlowJo software (version 7.6.1; TreeStar). Basophils were gated as SSC^low^/CD203c+/CD123+/HLA‐DR‐.^11^, ^17^ Basophil activation was expressed as %CD63+ basophils. Individuals with non‐responder basophils were defined as having %CD63+ basophils below 5% to anti‐IgE and response above 5% to fMLP, and were excluded from the R diagnostic analyses.

### 2.6 Oral food challenges

All study participants underwent OFC: infants <12 months of age underwent open incremental OFC and all other participants underwent DBPCFC. All negative DBPCFCs were followed by an age‐appropriate final open dose, for which both patients, parents and the clinical team were unblinded. Tables S1 and S2 show the dose regimens for OFC to BE and LCE, respectively. The outcome of the OFC was determined according to the Practall guidelines (Figure S1). DBPCFC were performed on one single day with placebo doses randomly interspersed between active doses, like what was done in the LEAP and EAT studies.^12^, ^13^ If a reaction to placebo occurred, a 2‐day DBPCFC was performed. Participants who reacted to placebo on the 2‐day DBPCFC, or who had a reaction to placebo but refused to have a 2‐day DBPCFC, or who did not complete the OFC or who had an indeterminate OFC were considered as not having an outcome and were excluded from the diagnostic analyses.

### 2.7 Statistical analyses

Categorial variables were represented as proportions and compared with chi‐square or Fisher's exact test, as appropriate. Quantitative variables were represented as median and interquartile range and compared with Mann–Whitney *U*‐test. ROC curve analyses were performed to assess the diagnostic accuracy of the various tests; simulation and resampling techniques were conducted to assess internal validity. Optimal cut‐offs were determined by the Youden index, compared with the outcome of OFC. Positive and negative cut‐offs were defined by the highest point in the ROC curve with 100% sensitivity and the lowest point in the same curve with 100% specificity. Sequential use of tests was considered for the patients whose results fell within positive and negative cut‐offs and for every sequence ended with OFC to clarify the allergic status of equivocal cases. All statistical evaluations were performed by SPSS version 27, internal validation was performed by numerical computation and resampling performed using the R software. Statistical tests were two tailed, and type‐I error rate was set to 5% (*α* = 0.05).

### 2.8 Sample size adequacy and statistical power assessment

A formal power calculation was conducted using the power.roc.test function from the pRoc package using the R software version 4.0.2. According to this calculation, a number of cases in the range of 50–60 and an equal number of non‐cases would result in a probability of false negative (type‐II error) from 1% to 5% (Statistical power in a range of 95%–99%) with an AUC of 0.75 or above, with a probability of false positive (type‐I error) in a range of 1%–5%. With the same number of cases and non‐cases and an AUC of 0.7, we calculated a statistical power in a range of 85% to 95%.

## 3 RESULTS

### 3.1 Baseline demographic and clinical characteristics of participants

**FIGURE 1:**
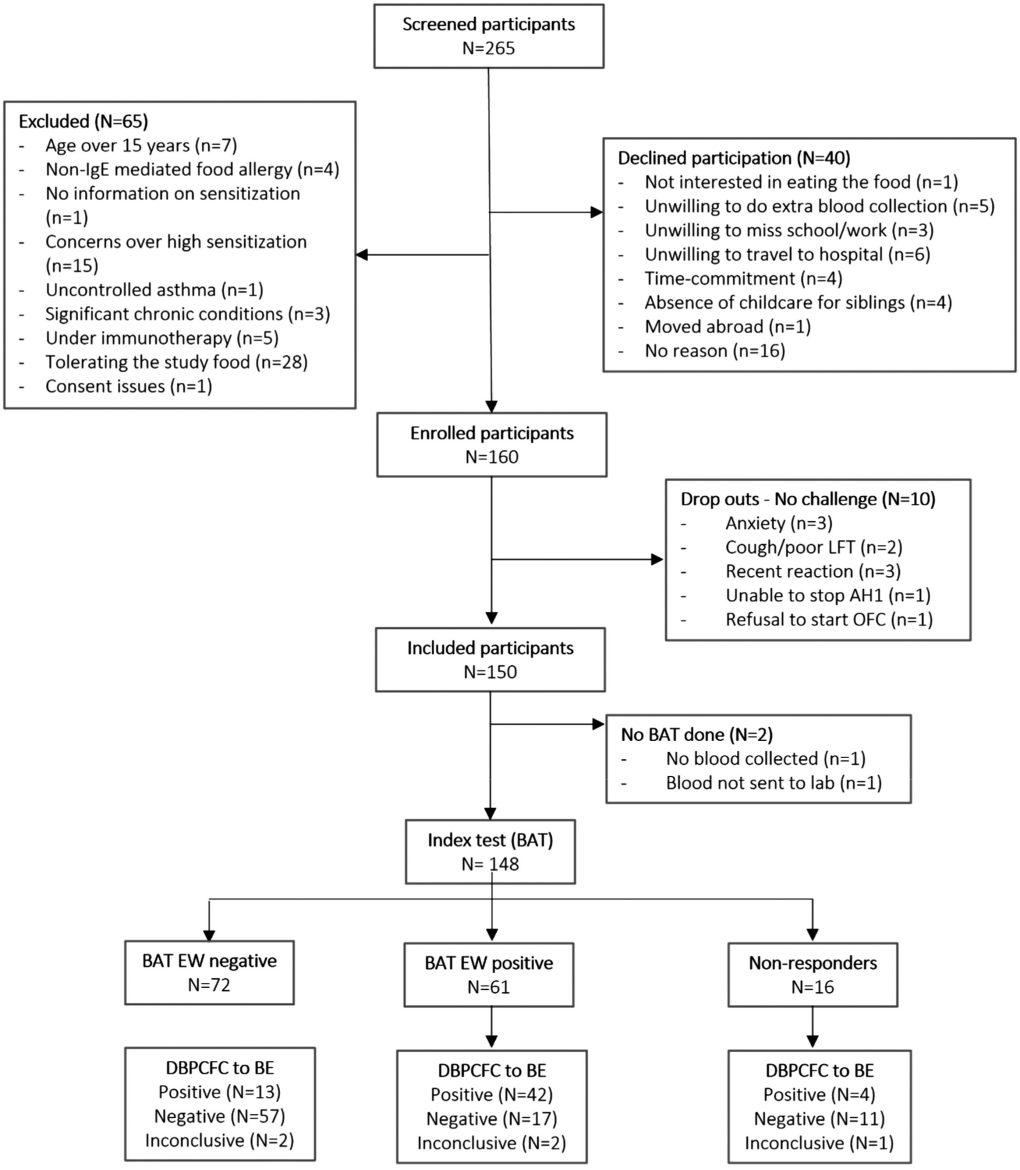
Flow diagram from the BAT2 egg study assessing the utility of the basophil activation test in predicting clinical reactivity to baked egg during DBPCFC. AH1, anti‐histamine; BAT, basophil activation test; BE, baked egg; DBPCFC, double‐blind placebo‐controlled food challenge; LFT, lung function test.

Out of the 265 children screened, 65 were not eligible, 40 declined participation and 10 refused to undergo OFC (Figure 1). One hundred and fifty children were recruited: 60 (40%) reacted to BE, 85 (57%) tolerated BE and 5 (3%) had inconclusive BE OFC. Two patients reacted after a placebo dose, and a 2‐day DBPCFC was undertaken. Table S3 describes the demographic and clinical characteristics of the study population. Out of the 60 children who reacted to BE, 13 (22% of positive OFC) required intra‐muscular adrenaline to treat their allergic symptoms and three of them (5% of positive OFC) required two doses of adrenaline. All patients responded well to treatment; three patients required an extended period of observation but none needed an inpatient hospital admission. Table S5 describes the outcomes of OFC in more detail. Out of the 85 children who passed their OFC to BE, 77 attended for a DBPCFC to LCE: 16 (21%) reacted and 61 (79%) tolerated LCE. Thus, in total, 60 (40%) patients were allergic to all forms of egg, 16 (11%) tolerated BE but reacted to LCE, 71 (47%) tolerated all forms of egg and 13 (9%) had indeterminate challenges or were lost to follow up.

### 3.2 Basophil activation test and specific IgE to egg white are the best tests to predict the outcome of baked egg and lightly cooked egg challenges

**FIGURE 2:**
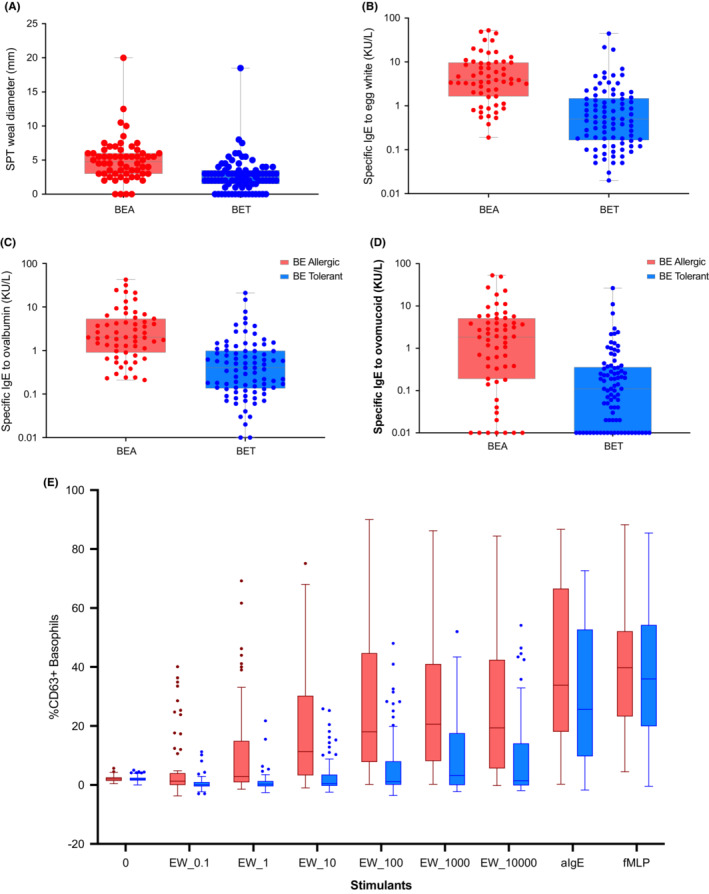
Skin prick test (A), sIgE (B, C, D) and BAT (E) results in baked egg allergic (BEA) and baked egg tolerant (BET) children as assessed by DBPCFC to baked egg.

**FIGURE 3:**
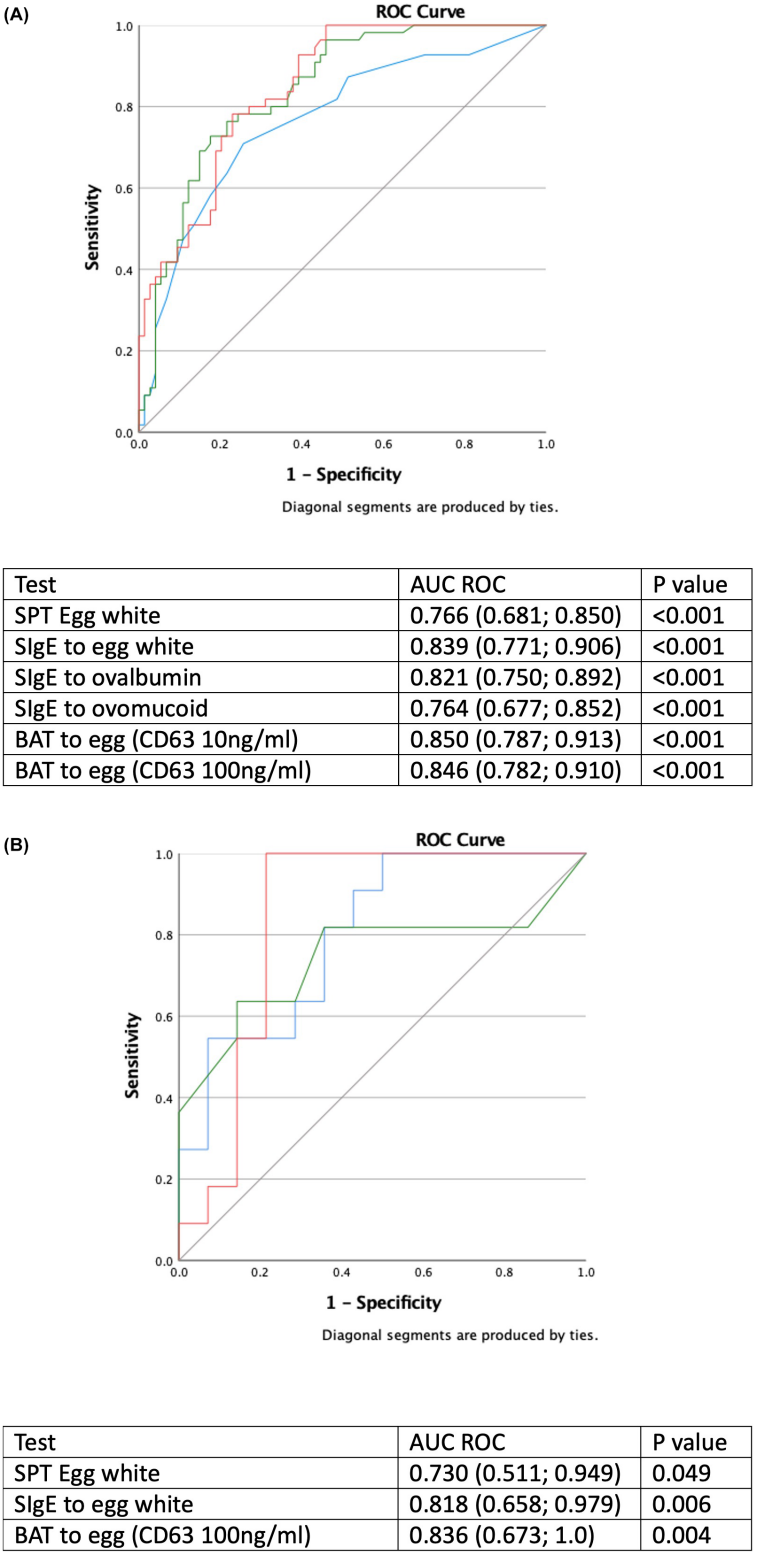
Receiver operator characteristic curves for best tests for each test modality (SPT to egg white in blue, specific IgE to egg white in green and BAT in red) for patients with results for all tests. (A) Children of all ages (*n* = 129, 55 allergic and 74 tolerant to baked egg). (B) Children younger than 2 years (*n* = 25, 11 allergic and 15 tolerant to baked egg).

The results of tests were compared between BE allergic and BE tolerant children (Figure 2; Table S4). The diagnostic performance of the various tests was determined in relation to the gold‐standard OFC. Among SPT, SPT to EW extract had the largest area under the ROC curve but was not statistically significantly different from SPT to raw egg or the BE slurry (Figure S2). Although the performance was similar, the diagnostic cut‐offs for SPT to raw egg were higher than the one for SPT to EW. We also assessed the diagnostic performance of the difference and the ratio between the results of SPT to EW and SPT to raw egg; however, neither the difference nor the ratio of SPT to EW and raw egg seemed useful (Figure S3). SIgE to EW had a larger area under the ROC than sIgE to ovalbumin or ovomucoid (Figure S4). In terms of BAT, 16 (10.8%) patients had non‐responder basophils. The stimulant that provided the largest area under the ROC curve for BAT was egg extract at 10 or 100 ng/mL using the activation marker CD63 (Figure S5). Figure 3 shows the ROC curve for the best test for each test modality, namely BAT to egg, specific IgE to EW and SPT to EW. BAT to egg and sIgE to EW were the tests with the best performance, both for BE and LCE allergies. All of the above‐mentioned ROC curves had AUC in agreement to resampling and simulation estimates pointing out for internal validity of our results.

### 3.3 Optimal, 100% sensitivity and 100% specificity diagnostic cut‐offs

Various cut‐offs were identified for SPT to EW, EW‐sIgE, OVA‐sIgE, OVM‐sIgE, BAT using CD63 and 100 ng/mL of egg extract and BAT using CD203c and 10 ng/mL of egg extract. These cut‐offs included the optimal cut‐off, positive and negative cut‐offs defined by 100% sensitivity and 100% specificity, respectively. The sensitivity, specificity, PPV, NPV, accuracy as well as number of true and false positives and true and false negatives are shown in Table 1 for BE for children of all ages and for children younger than 2 years. The younger children had lower cut‐offs for all tests, except for BAT. The performance of tests and the diagnostic cut‐offs for LCE allergy was similar to the ones determined for BE allergy (Table S6).

**TABLE 1:** Optimal, 100% sensitivity and 100% specificity cut‐offs for the various tests compared with the outcome of baked egg challenges.

Diagnostic tests	Cut‐off		AUC ROC	Sensitivity	Specificity	PPV	NPV	Diagnostic accuracy	TP/FP	TN/FN
**A. For all children**										
SPT Egg White Extract (mm)	100% S	0	0.5	100%	0%	43%	N/A	43%	55/74	0/0
	OPTIMAL	3	0.726	71%	74%	67%	78%	73%	39/19	55/16
	100% Sp	19	0.509	2%	100%	100%	58%	58%	1/0	74/54
SIgE Egg White (KU/L)	100% S	0.19	0.662	100%	32%	52%	100%	61%	55/50	24/0
	OPTIMAL	2.25	0.776	73%	82%	75%	80%	78%	40/13	61/15
	100% Sp	44.7	0.528	6%	100%	100%	59%	60%	3/0	74/52
SIgE to OVA (KU/L)	100% S	0.21	0.710	100%	42%	56%	100%	67%	55/43	31/0
	OPTIMAL	1.29	0.753	71%	80%	72%	79%	76%	39/15	59/16
	100% Sp	21.1	0.546	9%	100%	100%	60%	61%	5/0	74/50
SIgE to OVM (KU/L)	100% S	0.01	0.541	100%	8%	45%	100%	47%	55/68	6/0
	OPTIMAL	0.55	0.751	69%	81%	73%	78%	76%	38/14	60/17
	100% Sp	26.95	0.528	6%	100%	100%	59%	60%	3/0	74/52
BAT EW (%CD63 100 ng/mL)	100% S	2.2	0.771	100%	54%	62%	100%	74%	55/34	40/0
	OPTIMAL	9.4	0.776	78%	77%	78%	78%	77%	43/17	57/12
	100% Sp	48.8	0.618	67%	27%	100%	100%	64%	13/0	74/42
BAT EW (SI CD203c 10 ng/mL)	100% S	0.57	0.507	100%	1%	43%	100%	43%	55/73	1/0
	OPTIMAL	1.44	0.783	78%	78%	73%	83%	78%	43/16	58/12
	100% Sp	5.01	0.582	16%	100%	100%	62%	64%	9/0	74/46
**B. For children younger than 2 years**										
SPT Egg White Extract (mm)	100% S	N/A	–	–	–	–	–	–	–	–
	OPTIMAL	4	0.718	82%	60%	70%	75%	73%	7/3	12/4
	100% Sp	5	0.682	36%	100%	100%	68%	73%	4/0	15/7
SIgE Egg White (KU/L)	100% S	0.17	0.767	100%	53%	61%	100%	73%	11/7	8/0
	OPTIMAL									
	100% Sp	5.35	0.637	27%	100%	100%	65%	69%	3/0	15/8
BAT EW (%CD63 100 ng/mL)	100% S	2.3	0.867	100%	73%	73%	100%	85%	11/4	11/0
	OPTIMAL Youden									
	100% Sp	65.5	0.546	9%	100%	100%	60%	62%	1/0	15/10

Abbreviations: AUC ROC, area under the receiver operator characteristic curve; BAT, basophil activation test; EW, egg white; FN, false negatives; FP, false positives; NPV, negative predictive value; PPV, positive predictive value; S, sensitivity; sIgE, specific IgE; Sp, specificity; SPT, skin prick test; TN, true negatives; TP, true positives.

### 3.4 Combination of diagnostic tests enables optimisation of resources and improved patient outcomes

**FIGURE 4:**
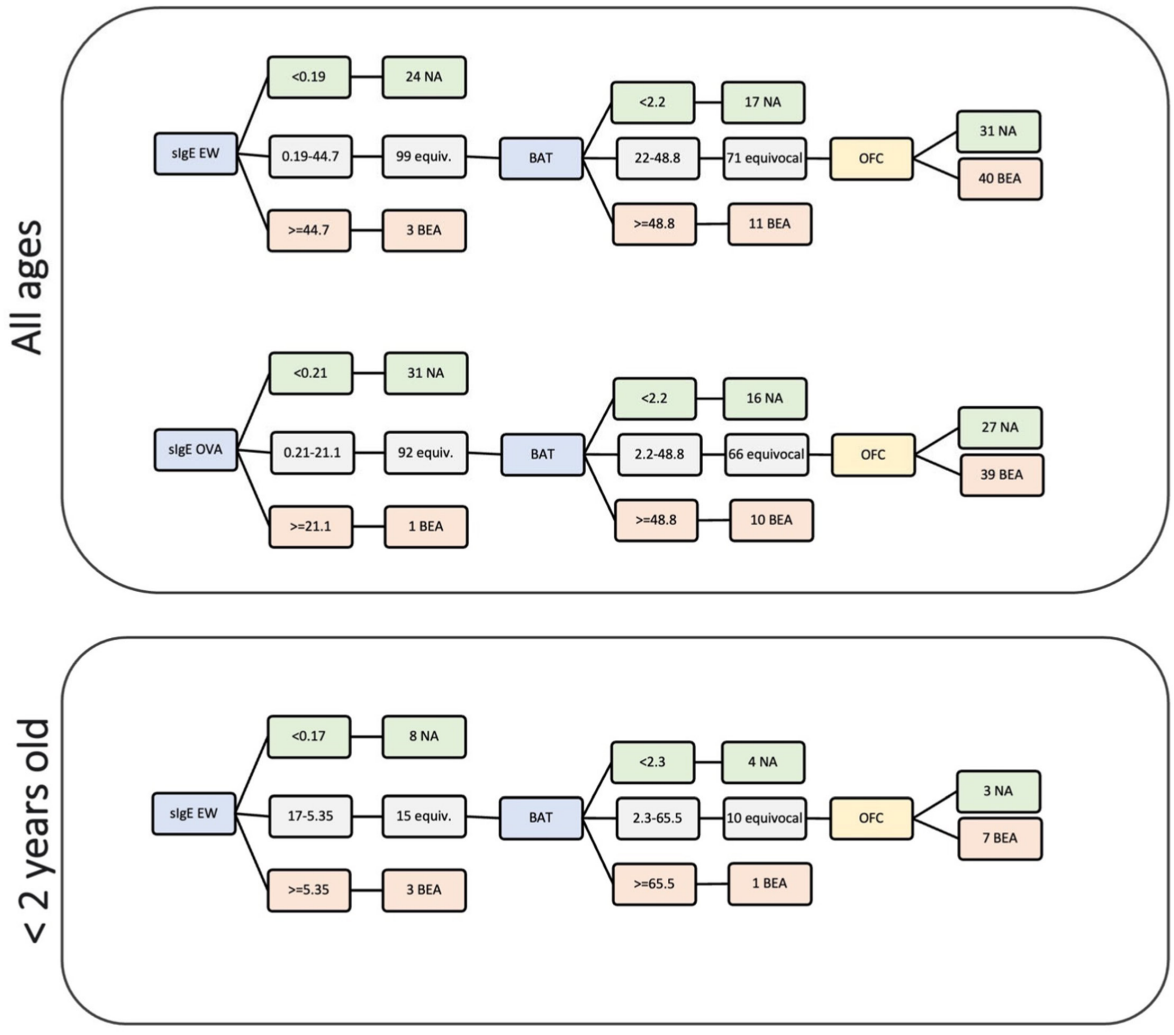
Sequential use of diagnostic tests to predict the outcome of challenges to baked egg with the basophil activation test (BAT) as a second step in the diagnostic process, after specific IgE to egg white (sIgE EW) or specific IgE to ovalbumin (sIgE OVA). Cut‐offs with 100% specificity (i.e. 44.7 KU/L for sIgE EW; 21.1 KU/L for sIgE OVA; 48.8% CD63+ basophils for BAT for children of all ages; and 5.35 KU/L for sIgE EW and 65.5% CD63+ basophils for BAT for children younger than 2 years) were used to confirm baked egg allergy (BEA); Cut‐offs with 100% sensitivity (i.e. 0.19 KU/L for sIgE EW; 0.21 KU/L for sIgE OVA; 2.2% CD63+ basophils for BAT for children of all ages; and 0.17 KU/L for sIgE EW and 2.3% CD63+ basophils for BAT for children younger than 2 years) were used to exclude allergy (NA, non‐allergic). Patients with results between these two cut‐offs would need an additional test (BAT) or an OFC—after OFC, patients were considered allergic or non‐allergic depending on the OFC outcome, positive or negative, respectively. Best combinations of tests for all ages and younger than 2 years age group are shown. This approach resulted in 100% accuracy.

Using 100% sensitivity and 100% specificity cut‐offs to identify non‐allergic and allergic children, respectively, and equivocal cases as the ones falling between cut‐offs, whom will need further testing and ultimately OFC to confirm the allergic status, avoids false‐positives and false‐negatives, resulting in 100% accuracy. Applying this approach to single tests followed by OFC, BAT was the test that significantly reduced the number of OFC (Table 2; Table S7) in both age groups (59% for all ages and 54% for younger than 2 years). Applying the same approach sequentially, with BAT as a second step in the diagnostic work‐up, allowed a similar reduction in patients requiring OFC and additionally a reduction in the number of BATs performed to about 70% considering children of all ages (Figure 4). The reduction in OFC and in the number of BATs was more marked in the younger age group with only 38% of children aged below 2 years needing an OFC and 58% being tested on BAT.

**TABLE 2:** Number of oral food challenges to baked egg required with individual tests using positive and negative cut‐offs.

Age group	Tests	NA	Equivocal	Allergic	OFC−	OFC+	%OFC	% OFC+
All ages (n = 129)	SPT EW	0	128	1	74	54	128/129 (99%)	54/128 (42%)
	sIgE EW	24	102	3	50	52	102/129 (79%)	52/102 (51%)
	sIgE OVA	31	93	5	43	50	93/129 (72%)	50/93 (54%)
	sIgE OVM	6	120	3	68	52	120/129 (93%)	52/120 (43%)
	**BAT‐CD63 EW100**	**40**	**76**	**13**	**34**	**42**	**76/129 (59%)**	**42/76 (55%)**
	sIgE EW → BAT	41	71	14	31	40	71/129 (55%)	40/71 (56%)
	**sIgE OVA → BAT**	**47**	**66**	**15**	**27**	**39**	**66/129 (51%)**	**39/66 (59%)**
	SPT EW → BAT	40	76	12	34	43	76/129 (59%)	43/76 (57%)
	SPT EW → sIgE EW	24	101	3	50	51	101/129 (78%)	51/101 (50%)
< 2 years (n = 26)	SPT EW	0	26	0	26	0	100.00%	0%
	sIgE EW	8	15	3	7	8	15/26 (58%)	8/15 (53%)
	BAT‐CD63 EW100	11	14	1	4	10	14/26 (54%)	10/14 (71%)
	**sIgE EW → BAT**	**12**	**10**	**8**	**3**	**7**	**10/26 (38%)**	**7/10 (70%)**

*Note*: Positive cut‐offs were used to confirm allergy; negative cut‐offs to exclude allergy and patients with results between cut‐offs would need an OFC. Tests or combination of tests highlighted in bold are the one with the best diagnostic performance.

Abbreviations: BAT, basophil activation test; EW, egg white; NA, non‐allergic; OFC, oral food challenge; sIgE, specific IgE; SPT, skin prick test.

## 4 DISCUSSION

Most egg allergic children tolerate BE.^3^ Being able to eat BE can improve children's diet and quality of life.^14^ However, previous studies suggest that tests currently available in clinical practice (i.e. SPT and sIgE) are not accurate predictors of clinical reactivity to BE,^7^ and it is difficult to provide advice about safe consumption of BE in egg allergic children without doing an OFC. OFC remain the reference standard for diagnosis, but they carry the risk of allergic reactions of unpredictable severity. In the BAT2 study, we included children who needed an OFC to clarify whether they were allergic to egg and compared the outcome of various tests with the outcome of DBPCFC to BE and LCE to better understand the diagnostic utility of the tests. We found that the test with the best diagnostic performance was BAT to egg followed by sIgE to EW. Using 100% sensitivity and 100% specificity cut‐offs to select non‐allergic and tolerant individuals, respectively, with equivocal cases being submitted to an additional test or OFC, ensured 100% diagnostic accuracy. With this approach, using a single test, BAT led to the greatest reduction in OFC, in all age groups. A similar approach, using BAT as a second step in the diagnostic process after sIgE to EW or OVA, reduced the number of BATs required with sustained reduction in OFC (compared to BAT only) and 100% diagnostic accuracy.

There are many strengths to underscore in this diagnostic study. First, the BAT2 study was rigorously designed according to the STARD guidelines.^15^ The BAT2 study is the largest study of its kind in which all participants were submitted to the reference standard OFC, namely DBPCFC (only infants had open OFC), and all diagnostic analyses were done in comparison with the outcome of OFC. All patients were carefully phenotyped, and in vitro tests were performed and analysed by a researcher blinded to all clinical features throughout the study. The proportion of egg allergic patients tolerating baked egg in our cohort was lower than previously reported,^16^ which is probably because this is a selected population of patients requiring OFC to BE. Egg allergic patients who tolerate an age‐appropriate amount of BE when they attend clinic are often recommended to continue with such consumption and are not referred for OFC; however, if we had included such patients, the overall proportion of BE tolerant patients in our study would have probably been higher. We performed diagnostic analyses not only for BAT but also for tests that are currently available to clinicians. We defined optimal cut‐offs and also 100% sensitivity and 100% specificity cut‐offs. Cut‐offs with high sensitivity are useful as screening tests to capture sensitisation. Cut‐offs with high specificity are useful to confirm food allergy, especially in the presence of a history of reaction.

Our results for BAT in the diagnosis of egg allergy, like previously for peanut allergy,^10^ support the incorporation of BAT in the diagnostic work‐up for egg allergy in clinical practice.^17^ BAT proved to be the best single test, allowing the best diagnostic accuracy and the greatest reduction in OFC. Adding BAT to existing tests could reduce allergic reactions during OFC. This is particularly important for paediatric populations. Indeed, BAT stood out from the other tests particularly in the younger than 2 years age group, which is the age group where the diagnosis of egg allergy is most often made for the first time. We speculate that the improved performance of BAT compared to IgE and SPT in the younger age group relates to its higher sensitivity to the allergen compared to allergen‐specific IgE, similar to what has been described for non‐allergic rhinitis^18^ and for peanut allergy^19^; and to the shorter half‐life of basophils and IgE compared with mast cells in the tissue, which makes BAT more susceptible to change and thus to reflect the current clinical phenotype. Considering all ages, sIgE to EW was the second‐best test. As sIgE is cheaper and easier to perform than BAT, we thought that including sIgE as a first step and BAT as a second step in the diagnostic work‐up could reduce the number of BAT required, improve feasibility (as BAT needs to be done on fresh cells hours after blood collection) and cost of the diagnostic process overall, and still reduce OFC. However, using two blood tests sequentially with BAT, which needs to be done soon after blood collection, being done as a second step, may require 2 blood draws which may be logistically challenging or not accepted by some families. An alternative could be to do BAT in all participants and then add sIgE as second test, as previously suggested by another group in a sesame study.^20^ Applying 100% sensitivity and 100% specificity cut‐offs, rather than optimal cut‐offs, improved the performance of the diagnostic work‐up overall, aiming for 100% diagnostic accuracy for all patients. Our work represents an authentic contribution to the clinical choice of allergy tests when investigating BE allergy in children; a target population where egg represents a valid and economic source of high‐quality nutrients.^21^, ^22^ The cut‐offs defined using the outcome of LCE challenges being similar to the ones defined based on BE challenges probably results from the small number of patients who reacted to LCE among BE tolerant patients.

Importantly, the BAT2 study improved our understanding of the diagnostic performance of the tests we already have available in clinic, such as SPT and sIgE, and of how we can optimise the results provided to improve patients' outcomes. Ongoing validation will confirm whether the diagnostic cut‐offs we defined are generalisable to other centres in the United Kingdom and the world. Some of our findings for SPT and sIgE are consistent with previous studies, for instance, the lack of added value of testing patients to both EW extract and the actual food, either as raw egg or BE.^23^, ^24^ The difference or the ratio between the results of these two tests were less predictive of BE allergy than each result individually. SPT to raw egg had a similar diagnostic performance to SPT to EW extract, albeit with higher cut‐offs. These results are surprising as one would expect the SPT to the form eaten in the OFC to be more informative and the discrepancy between different levels of processing of egg to reflect intermediate degrees of tolerance between baked and non‐baked egg. The performance of SPT overall was slightly disappointing and may result from the fact that patients with large SPT tended to not be referred for OFC. In that case, SPT would have been used as a screening test and worked less well as a confirmatory test in a population selected largely based on SPT. Conversely, other findings contrasted with previous studies, such as the improved predictive value of sIgE to OVA compared to sIgE to OVM.^8^ A possible explanation for the superiority of OVA‐sIgE in our dataset could be that, whilst OVM is a thermostable protein and therefore present in BE,^25^ most patients are sensitised to multiple egg allergens which are also present in BE, including OVA^26^; thus, the effect of sensitisation to the single allergen OVM may be lost when assessing the effect of a polyclonal response present in patients' serum. Furthermore, another study looking at IgE to egg components did not report advantage of OVM‐sIgE,^27^ and a more recent study of IgE epitopes on peptides from OVA and OVM showed that a combination of peptides from the two allergens was the best predictor or reactivity to BE.^28^

Children were advised to avoid baked egg for 2 days prior to the challenge given due to the possible interference of egg allergens in circulation with the outcome of the OFC and/or the BAT. This was decided based on the findings of the peanut oral immunotherapy study by Thyagarajan et al.^29^ which showed suppression of basophils' response to not only peanut but also bystander allergen, such as egg and IgE‐mediated controls, possibly due to basophil anergy induced by peanut consumption. However, this short period of avoidance in the Bat2 study does not seem to have affected basophil response, given that egg consumption was associated with lower basophil response in both allergic and tolerant subjects,^30^ despite the 2‐day period of avoidance in all study participants. Similar to previous studies,^10^, ^31^ 11% of tested subjects had non‐responder basophils, that is basophils that did not respond to allergen or the IgE‐mediated positive control but only to the non‐IgE mediated positive control fMLP, and most (69%) of these were tolerant to egg. The non‐responder phenotype can be transient^31^ and it is worth repeating BAT, if possible. If this is not possible, in these cases, the diagnosis needs to be supported by other tests, such as SPT, specific IgE and OFC, given that the BAT result is uninterpretable.

Despite the many strengths, we must acknowledge some potential limitations of our study. First, the fact that study participants were referred to the study based on their need for OFC, that some patients were excluded due to concerns over high IgE sensitisation and high likelihood of reaction during the OFC, and that other patients declined participation or ended up not undergoing OFC for a variety of reasons may have created some selection bias. However, these aspects also reflect the relevance of the selected population to the research question and to day‐to‐day clinical practice. Moreover, our study population is based on sequential recruitment which guarantee an acceptable degree of randomness of the sample and is obtained by numerous independent clinicians which guarantee the representativeness of the target population. Second, we cannot exclude that the sample size adopted may have limited our study with some false negative results. However, we showed that lack of statistical power affects our study only for AUC below the value of 0.7, a value that is not of clinical interest. Third, the data for the age group younger than 2 years are valuable, as this is the age group when the diagnosis of egg allergy is most often made for the first time. The diagnostic performance of BAT in this age group was impressive; however, the number of participants younger than 2 is limited to 25 children. Fourth, like any test, BAT is not absolute and there are patients with discrepant results when comparing the outcome of BAT and DBPCFC, as highlighted in Figure 1 (e.g. using optimal cut‐off and a dichotomous outcome, 25% of the BAT non‐responders reacted to an OFC, 28% of those with positive BAT had a negative OFC and 18% of those with negative BAT had a positive OFC). This is why the use of cut‐offs can be helpful and particularly the use of 100% sensitivity and 100% specificity cut‐offs with sequential use of tests can be helpful and reach very high overall diagnostic accuracy. Finally, our estimates were validated internally but not externally. We are currently in the process of validating the diagnostic performance of tests and their cut‐offs in a new independent cohort prospectively recruited as part of a multicentre study in the United Kingdom, the BAT Impact study (NCT05309772). Furthermore, specific meta‐analysis of diagnostic test accuracy are needed to confirm the reported results and improved access and cost‐effectiveness studies are needed to further support the integration of BAT in clinical practice. Once BAT is available, education of healthcare professionals on the use and interpretation of BAT is important to facilitate its implementation and appropriate clinical application.

In summary, overall, BAT to egg was the best test to predict BE and LCE allergies and, out of tests currently available in clinical practice, the best test was sIgE to EW. SPT had a poorer diagnostic performance than anticipated. The addition of BAT to egg, for patients with equivocal results for sIgE to EW, added precision to the diagnosis and reduced the number of patients undergoing OFC compared to what we would have needed to clarify all equivocal cases, making the diagnostic process safer and more comfortable for patients. This greater precision and convenience can enable a more rigorous and accessible diagnostic approach and reduce over‐diagnosis due to over‐reliance on SPT or sIgE tests alone and to limited capacity for OFC. Further external validation is currently underway in a UK multicentre study (NCT05309772) to confirm the generalisability of our findings.

## AUTHOR CONTRIBUTIONS

AMM, SR, RF, IB, MK, GdT and AFS performed study procedures related to patient recruitment and cared for study participants. MK and ZJ performed and analysed the basophil activation test. FH and AFS designed the food frequency questionnaires and oral food challenge protocols. CR performed the statistical analyses. GdT and GL funded some of additional support staff in the Seal unit and critically reviewed the study protocol. AFS designed the study protocol and acted as chief investigator for the study, obtained and managed the research funding, supervised data acquisition, data management and data analyses, and wrote the first version of the manuscript. All authors critically reviewed the manuscript and approved its final version.

## FUNDING INFORMATION

This study was funded by the Medical Research Council through MRC Clinician Scientist Fellowship MR/M008517/1 and MRC Transition Fellowship MR/T032081/1 awarded to A.F.Santos.

## CONFLICT OF INTEREST STATEMENT

Dr Radulovic reports salary support from grants from National Institute of Allergy and Infectious Diseases (NIAID, NIH). Dr Lack reports grants from National Institute of Allergy and Infectious Diseases (NIAID, NIH), other from Food Allergy & Research Education (FARE), other from MRC & Asthma UK Centre, other from UK Dept of Health through NIHR, other from National Peanut Board (NPB), other from The Davis Foundation, during the conduct of the study; shareholder in DBV Technologies, and Mighty Mission Me, personal fees from Novartis, personal fees from Sanofi‐Genyzme, personal fees from Regeneron, personal fees from ALK‐Abello, personal fees from Lurie Children's Hospital, outside the submitted work. Dr Du Toit reports grants from National Institute of Allergy and Infectious Diseases (NIAID, NIH), Food Allergy & Research Education (FARE), MRC & Asthma UK Centre, UK Dept of Health through NIHR, Action Medical Research and National Peanut Board. Scientific Advisory Board member Aimmune. Investigator on pharma‐sponsored allergy studies (Aimmune, and DBV Technologies). Scientific advisor to Aimmune, DBV and Novartis. Dr. Santos reports grants from Medical Research Council (MR/M008517/1; MC/PC/18052; MR/T032081/1), Food Allergy Research and Education (FARE), the Immune Tolerance Network/National Institute of Allergy and Infectious Diseases (NIAID, NIH), Asthma UK (AUK‐BC‐2015‐01), BBSRC, Rosetrees Trust and the NIHR through the Biomedical Research Centre (BRC) award to Guy's and St Thomas' NHS Foundation Trust, during the conduct of the study; personal fees from Thermo Scientific, Nutricia, Infomed, Novartis, Allergy Therapeutics, Buhlmann, as well as research support from Buhlmann and Thermo Fisher Scientific through a collaboration agreement with King's College London. The other authors have nothing to disclose.

## Supporting information


Data S1

## Data Availability

The data that support the findings of this study are available on request from the corresponding author. The data are not publicly available due to privacy or ethical restrictions.
