# Supplementary material for: Diagnostic utility of allergy tests to predict baked egg and lightly cooked egg allergies compared to double‐blind placebo‐controlled food challenges
Source: Allergy. 2023 Jul 7;78(9):2510–22. doi: 10.1111/all.15797 (PMC10790315; doi:10.1111/all.15797)
Supplement: Supplementary file 1 — Data S1 [file ALL-78-2510-s001.docx]

**Online Supplementary Material**

**E-Methods**

**Exclusion criteria of the BAT2 study**

Exclusion criteria were: clinically significant chronic illness other than atopic diseases; previous history of severe life-threatening reaction to egg with documented decrease in oxygen saturation (<90%), hypotension (≥20% reduction in systolic blood pressure) and/or admission to intensive care; unwillingness to undergo a diagnostic OFC; contra-indication for OFC, namely uncontrolled atopic diseases (e.g. eczema, asthma, rhinitis), chronic medical conditions that pose significant risk in the event of anaphylaxis or treatment of anaphylaxis (e.g. cardiac disease, severe lung disease, pregnancy), inability to discontinue medications that might interfere with assessment or safety (e.g. antihistamines, β-agonists, β-blockers, NSAIDs, ACE inhibitor, antacids), recent (within 7-14 days) treatment with systemic steroids or prolonged high-dose systemic steroids or immunosuppressants, undergoing treatment with omalizumab, food allergen immunotherapy or other systemic immunomodulatory treatment; inability to stop anti-histamines prior to SPT. In the case of uncontrolled atopic diseases or recent treatment with systemic corticosteroids, potential participants can have their eligibility criteria revised at a later time-point and may be considered eligible once co-existent atopic conditions are controlled or after a wash-out period of systemic steroids, respectively.

**Table E1.** Dose regimens in grams of protein for baked egg challenges. *The initial doses will be given only in patients considered to be high-risk (HR). **Cumulative dose does not include the High-Risk doses

| **Age** | | **6-12 months** | **1-3 years** | **4-10 years** | **11-16 years** |
| --- | --- | --- | --- | --- | --- |
| Type of challenge | | Open | DBPCFC | DBPCFC | DBPCFC |
| Number of placebo doses randomly interspersed | | 0 | 1 | 1 | 1 |
| Active doses  (g) | High Risk Dose 1* | 0.003 | 0.003 | 0.003 | 0.003 |
|  | High Risk Dose 2* | 0.01 | 0.01 | 0.01 | 0.01 |
|  | Dose 1 | 0.03 | 0.03 | 0.03 | 0.03 |
|  | Dose 2 | 0.1 | 0.1 | 0.1 | 0.1 |
|  | Dose 3 | 0.2 | 0.3 | 0.3 | 0.3 |
|  | Dose 4 | 0.4 | 0.6 | 0.6 | 0.6 |
|  | Open dose | 0.8 | 1.0 | 1.5 | 2.5 |
|  | **Cumulative protein dose**** | **1.53** | **2.03** | **2.53** | **3.53** |
|  | Total number of muffin/cookies | 1.0 | 1.3 | 1.6 | 2.2 |

**Table E2.** Dose regimens in grams of protein for DBPCFC to lightly cooked egg according to the different age groups.*The initial doses will be given only in patients considered to be high-risk (HR). **Cumulative dose does not include the High Risk doses.

| Age | | 6-12 months | 1-3 years | 4-10 years | 11-16 years |
| --- | --- | --- | --- | --- | --- |
| Food | | LIGHTLY COOKED EGG | | | |
| Type of challenge | | Open | DBPCFC | DBPCFC | DBPCFC |
| Number of placebo doses randomly interspersed | | 0 | 1 | 1 | 1 |
| Active doses  (g) | High Risk Dose 1* | 0.003 | 0.003 | 0.003 | 0.003 |
|  | High Risk Dose 2* | 0.01 | 0.01 | 0.01 | 0.01 |
|  | Dose 1 | 0.03 | 0.03 | 0.03 | 0.03 |
|  | Dose 2 | 0.1 | 0.1 | 0.1 | 0.1 |
|  | Dose 3 | 0.3 | 0.3 | 0.3 | 0.3 |
|  | Dose 4 | 1.0 | 1.0 | 1.0 | 1.0 |
|  | Dose 5 |  |  |  |  |
|  | Dose 6 |  |  |  |  |
|  | Open dose 1 | 2.0 | 2.0 | 2.0 | 2.0 |
|  | Open dose 2 | 3.0 | 4.0 | 5.0 | 6.0 |
|  | **Cumulative protein dose**** | **6.43** | **7.43** | **8.43** | **9.43** |
|  | Total number of 50g medium eggs | 0.9 | 1.0 | 1.2 | 1.3 |

**Table E3.** Baseline demographic and clinical characteristics of participants. Number and percentage are indicated for qualitative variables and median and interquartile range are represented for quantitative variables. P values are indicated for the comparison between baked egg allergic and baked egg tolerant using ^a^Mann-Whitney U test

| **Demographic and clinical characteristics** | **BE allergic (n=60)** | **BE tolerant (n=85)** | **P value** |
| --- | --- | --- | --- |
| Age (years) | 5.8 (2.6; 8.9) | 4.5 (2.4; 8.5) | 0.286 |
| Gender (% females) | 26 (43%) | 34 (40%) | 0.734 |
| Ethnicity |  |  |  |
| - White | 39 (65%) | 50 (59%) |  |
| - Black | 6 (10%) | 6 (7.1%) | 0.442 |
| - Asian | 4 (7%) | 8 (9%) |  |
| - Chinese | 3 (5%) | 1 (1.2%) |  |
| - Mixed | 5 (8%) | 14 (17%) |  |
| - Other | 3 (5%) | 6 (7%) |  |
| History of allergic reaction to egg (%)   - Baked egg - Whole egg - Raw egg | 26 (43%)  48 (80%)  30 (50%) | 37 (44%)  63 (74%)  40 (47%) | 1.0  0.434  0.739 |
| History of consumption of baked egg (%) | 9 (17%) | 50 (62%) | **<0.001** |
| Atopic eczema (%) | 48 (80%) | 72 (85%) | 0.301 |
| Other food allergies (%) | 46 (77%) | 56 (66%) | 0.130 |
| Allergic rhinitis (%) | 25 (42%) | 31 (37%) | 0.322 |
| Asthma (%) | 24 (40%) | 20 (24%) | **0.044** |

**Table E4.** Diagnostic markers in baked egg allergic and baked egg tolerant. Median and interquartile range are represented. P values are indicated for the comparison between baked egg allergic and baked egg tolerant using ^a^Mann-Whitney U test

| **Diagnostic markers** | **BE allergic (n=60)** | **BE tolerant (n=85)** | **P value** |
| --- | --- | --- | --- |
| SPT to egg white extract (mm) | 5 (3; 6) | 3 (2; 4) | **<0.001**^a^ |
| SPT to raw egg (mm) | 9 (8; 11) | 6 (4;8) | **<0.001**^a^ |
| SPT to baked egg slurry (mm) | 3 (0; 4) | 0 (0; 2) | **<0.001**^a^ |
| Difference RE and EW SPT | 4 (3; 7) | 3 (2; 5) | **0.013** |
| Ratio RE/EW SPT | 1.91 (1.44; 2.51) | 2.20 (1.72; 3.0) | 0.119 |
| Total IgE (kU_A_/L) – n=59 vs 84 | 267 (87; 1101) | 169 (52; 1016) | 0.140^a^ |
| Specific IgE to Egg (kU_A_/L) | 3.81 (1.57; 9.40) | 0.52 (0.18; 1.47) | **<0.001**^a^ |
| Specific IgE to Egg White (kU_A_/L) | 3.86 (1.64; 9.66) | 0.50 (0.17; 1.49) | **<0.001**^a^ |
| Specific IgE to Gal d 1 (kU_A_/L) | 1.84 (0.19; 5.11) | 0.11 (0.01; 0.36) | **<0.001**^a^ |
| Specific IgE to Gal d 2 (kU_A_/L) | 2.02 (0.90; 5.38) | 0.40 (0.14; 0.99) | **<0.001**^a^ |
| Specific IgG4 to Egg (mg/L) | 0.22 (0.09; 0.94) | 0.39 (0.05; 2.14) | 0.468 |
| Specific IgG4 to Egg White (mg/L) | 0.16 (0.05; 0.77) | 0.28 (0.03; 1.43) | 0.422 |
| Specific IgG4 to Gal d 1 (mg/L) | 0.04 (0.01; 0.28) | 0.01 (0.01; 0.56) | 0.468 |
| Specific IgG4 to Gal d 2 (mg/L) | 0.17 (0,05; 0.78) | 0.31 (0.03; 1.84) | 0.336 |
| IgG4/IgE ratio to egg | 26.7 (6.4; 94.8) | 241.5 (51.2; 1226.0) | **<0.001**^a^ |
| IgG4/IgE ratio to egg white | 14.5 (4.2; 69.0) | 163.7 (38.9; 951.0) | **<0.001**^a^ |
| IgG4/IgE ratio to Gal d 1 | 12.2 (2.2; 197.7) | 208.3 (23.8; 272.3) | **<0.001**^a^ |
| IgG4/IgE ratio to Gal d 2 | 28.0 (6.2; 133.6) | 189.4 (36.8; 1229.9) | **<0.001**^a^ |
| BAT to egg white extract at 100ng/ml  (%CD63+ Basophils) | 18.03 (7.75; 44.73) | 1.08 (0.02; 8.02) | **<0.001**^a^ |
| BAT to egg white extract at 10ng/ml  (SI CD203c) | 2.26 (1.41; 4.08) | 1.09 (1.0; 1.29) | **<0.001**^a^ |
| BAT with no stimulation  (%CD63+ Basophils) | 2.04 (1.40; 2.58) | 1.90 (1.46; 2.47) | 0.413^a^ |
| BAT to anti-IgE  (%CD63+ Basophils) | 33.81 (18.04; 66.63) | 25.57 (9.74; 52.74) | **0.024^a^** |
| BAT to fMLP  (%CD63+ Basophils) | 39.77 (23.24; 52.14) | 35.94 (19.90; 54.30) | 0.804^a^ |

**Table E5.** Outcome of oral food challenges to baked egg in the BAT2 study.

| Outcome of oral food challenge to baked egg | Symptoms developed during the oral food challenge | | Cumulative dose of baked egg tolerated (in grams) | Treatment provided during the oral food challenge |
| --- | --- | --- | --- | --- |
| POSITIVE | Erythematous rash | 14 (23%) | 0.13  (0.03; 0.44) | Adrenaline  13 (22%)  Anti-histamine (systemic)  60 (100%)  Corticosteroids (systemic)  10 (17%)  Salbutamol (inhaled)  7 (12%)  Oxygen  7 (12%)  Intravenous fluids  1 (2%) |
|  | Pruritus | 19 (32%) |  |  |
|  | Urticaria/angioedema | 24 (40%) |  |  |
|  | Rash | 10 (17%) |  |  |
|  | Sneezing / itching | 30 (50%) |  |  |
|  | Wheezing | 5 (8%) |  |  |
|  | Laryngeal symptoms | 16 (27%) |  |  |
|  | Subjective gastrointestinal complaints | 35 (58%) |  |  |
|  | Objective gastrointestinal complaints | 14 (23%) |  |  |
|  | Cardiovascular / Neurologic | 3 (5%) |  |  |
| NEGATIVE | Nil | | 2.53  (2.03; 2.54) | Nil |
| INCONCLUSIVE | 1 Challenge stopped due to anxiety  3 Children refused to eat (1 child refused dose 4 and 2 children refused the final dose)  1 Child had mild abdominal pain during final dose. | | | |

**Table E6.** Diagnostic cut-offs for the various tests compared with the outcome of challenges to loosely cooked egg: optimal, 100% sensitivity and 100% specificity.

| **Diagnostic tests** | **Cut-off** | | **AUC ROC** | **Sensitivity** | **Specificity** | **PPV** | **NPV** | **Diagnostic accuracy** | **TP/FP** | **TN/FN** |
| --- | --- | --- | --- | --- | --- | --- | --- | --- | --- | --- |
| SPT EW | 100% S | - | - | - | - | - | - | - | - | - |
|  | OPTIMAL | 3 | 0.710 | 62% | 80% | 80% | 62% | 70% | 43 / 11 | 43 / 26 |
|  | 100% Sp | 19 | 0.507 | 1% | 100% | 100% | 44% | 45% | 1 / 0 | 54 / 68 |
| sIgE EW | 100% S | 0.07 | 0.565 | 100% | 13% | 60% | 100% | 62% | 69 / 47 | 7 / 0 |
|  | OPTIMAL | 1.88 | 0.732 | 67% | 80% | 81% | 65% | 72% | 46 / 11 | 43 / 23 |
|  | 100% Sp | 44.7 | 0.522 | 4% | 100% | 100% | 45% | 46% | 3 / 0 | 54 / 66 |
| sIgE OVM | 100% S | 0.01 | 0.556 | 100% | 11% | 59% | 100% | 61% | 69 / 48 | 6 / 0 |
|  | OPTIMAL | 0.55 | 0.723 | 59% | 85% | 84% | 62% | 71% | 41 / 8 | 46 / 28 |
|  | 100% Sp | 26.95 | 0.522 | 4% | 100% | 100% | 45% | 46% | 3 / 0 | 54 / 66 |
| sIgE OVA | 100% S | 0.04 | 0.547 | 100% | 9% | 59% | 100% | 60% | 69 / 49 | 5 / 0 |
|  | OPTIMAL | 1.28 | 0.728 | 62% | 83% | 83% | 63% | 72% | 43 / 9 | 45 / 26 |
|  | 100% Sp | 18.9 | 0.536 | 7% | 100% | 100% | 46% | 0.479 | 5 / 0 | 54 / 64 |
| BAT CD63 EW 100 | 100% S | 0 | 0.593 | 100% | 19% | 61% | 100% | 64% | 69 / 44 | 10 / 0 |
|  | OPTIMAL | 9.4 | 0.732 | 67% | 80% | 81% | 65% | 72% | 46 / 11 | 43 / 23 |
|  | 100% Sp | 48.8 | 0.594 | 19% | 100% | 100% | 49% | 0.594 | 13 / 0 | 54 / 56 |

**Table E7.** Number of oral food challenges to lightly cooked egg required with individual tests using positive and negative cut-offs. Positive cut-offs were used to confirm allergy, negative cut-offs to exclude allergy and patients with results between cut-offs would need an OFC.

| TESTS | NA | Equivocal | Allergic | OFC- | OFC+ | %OFC | %OFC+ |
| --- | --- | --- | --- | --- | --- | --- | --- |
| SPT EW | 0 | 123 | 0 | 54 | 69 | 123/123 (100%) | 69/123 (56%) |
| sIgE EW | 7 | 113 | 3 | 47 | 66 | 113/123 (92%) | 66/113 (58%) |
| sIgE OVM | 6 | 114 | 3 | 48 | 66 | 114/123 (93%) | 66/114 (58%) |
| sIgE OVA | 5 | 113 | 5 | 49 | 64 | 113/123 (92%) | 64/113 (57%) |
| BAT-CD63 EW100 | 10 | 100 | 13 | 44 | 56 | 100/123 (81%) | 56/100 (56%) |
| sIgE EW -> BAT | 7 | 94 | 14 | 39 | 55 | 94/123 (76%) | 55/94 (59%) |
| sIgE OVA -> BAT | 5 | 94 | 15 | 40 | 54 | 94/123 (76%) | 54/94 (57%) |

**Figure E1.** Criteria for stopping the oral food challenge followed the Practall guidelines.


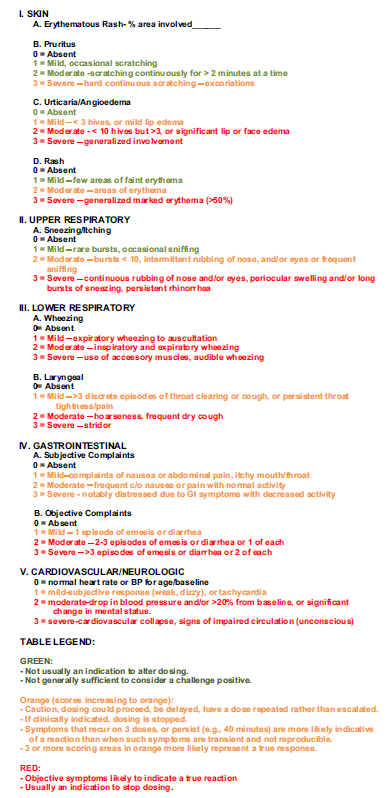


**Figure E2.** Receiver operator characteristic curve for different skin prick tests compared with the outcome of baked egg challenges.


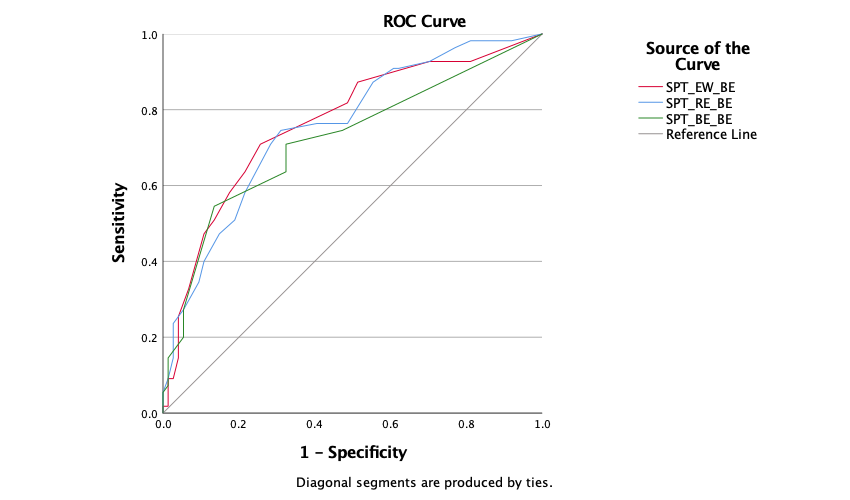


| Test Result Variable(s) | Area | 95% Confidence Interval | |
| --- | --- | --- | --- |
|  |  | Lower Bound | Upper Bound |
| SPT_EW_BE | .766 | .681 | .850 |
| SPT_RE_BE | .756 | .672 | .840 |
| SPT_BE_BE | .721 | .629 | .813 |

**Figure E3.** Receiver operator characteristic curve for difference and ratio between SPT to raw egg and SPT to egg white to predict outcome of DBPCFC to baked egg.


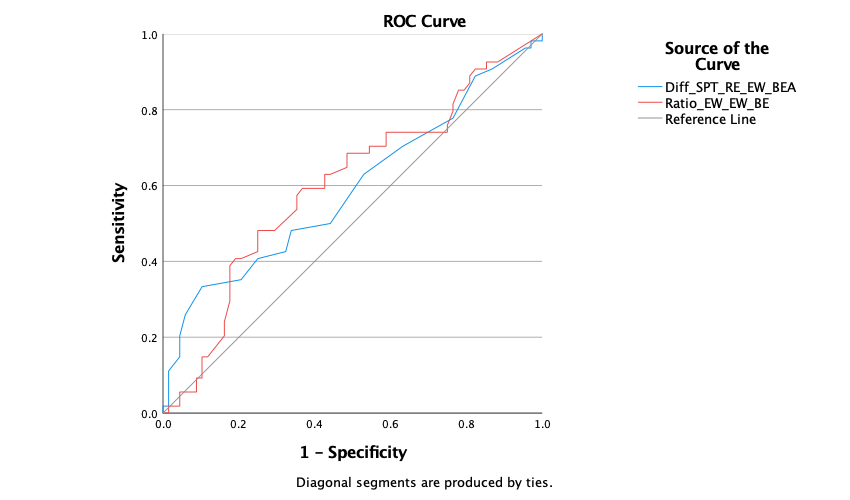


| Test Result Variable(s) | Area | Asymptotic 95% Confidence Interval | |
| --- | --- | --- | --- |
|  |  | Lower Bound | Upper Bound |
| Diff_SPT_RE_EW_BEA | .591 | .487 | .695 |
| Ratio_EW_EW_BE | .601 | .499 | .703 |

**Figure E4.** Receiver operator characteristic curve for different specific IgE tests compared with the outcome of baked egg challenges.

.


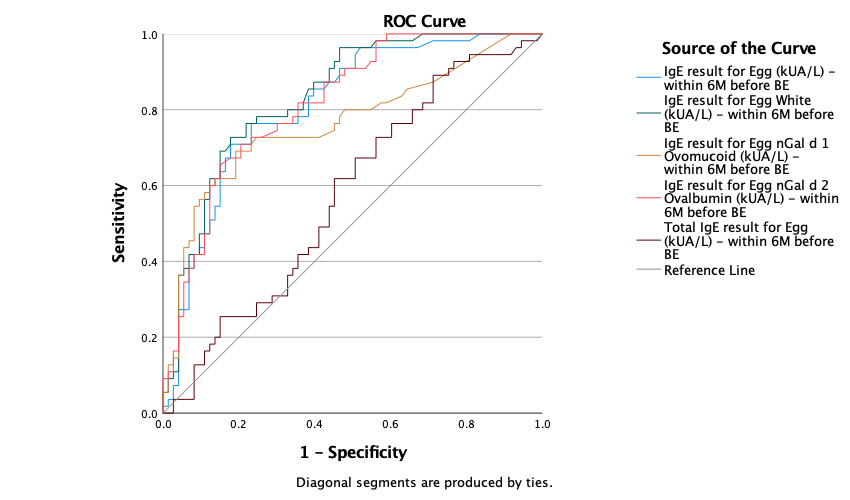


| Test Result Variable(s) | Area | 95% Confidence Interval | |
| --- | --- | --- | --- |
|  |  | Lower Bound | Upper Bound |
| IgE to Egg (kUA/L) | .811 | .737 | .886 |
| IgE to Egg White (kUA/L) | .836 | .768 | .905 |
| IgE to nGal d 1 Ovomucoid (kUA/L) | .764 | .676 | .851 |
| IgE to nGal d 2 Ovalbumin (kUA/L) | .818 | .747 | .890 |
| Total IgE (kUA/L) | .574 | .475 | .673 |

**Figure E5.** Receiver operator characteristic curve for different concentrations of two preparations of egg compared with the outcome of baked egg challenges: egg white extract (EW) and baked egg white (BE) in the basophil activation test using CD63 or CD203c as activation marker.

#### CD63 and egg white extract


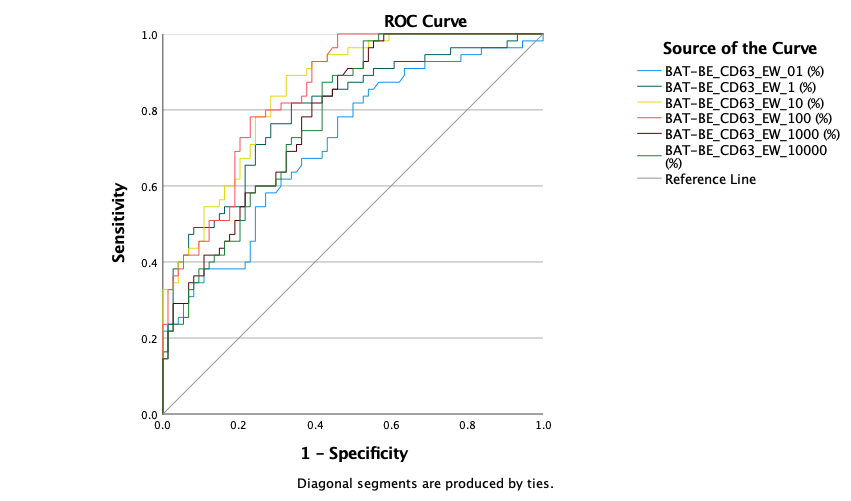


| Test Result Variable(s) | Area | 95% Confidence Interval | |
| --- | --- | --- | --- |
|  |  | Lower Bound | Upper Bound |
| CD63_EW_01 (%) | .714 | .624 | .803 |
| CD63_EW_1 (%) | .795 | .717 | .873 |
| CD63_EW_10 (%) | .850 | .787 | .913 |
| CD63_EW_100 (%) | .846 | .782 | .910 |
| CD63_EW_1000 (%) | .784 | .708 | .860 |
| CD63_EW_10000 (%) | .779 | .702 | .856 |

1. CD63 and baked egg white


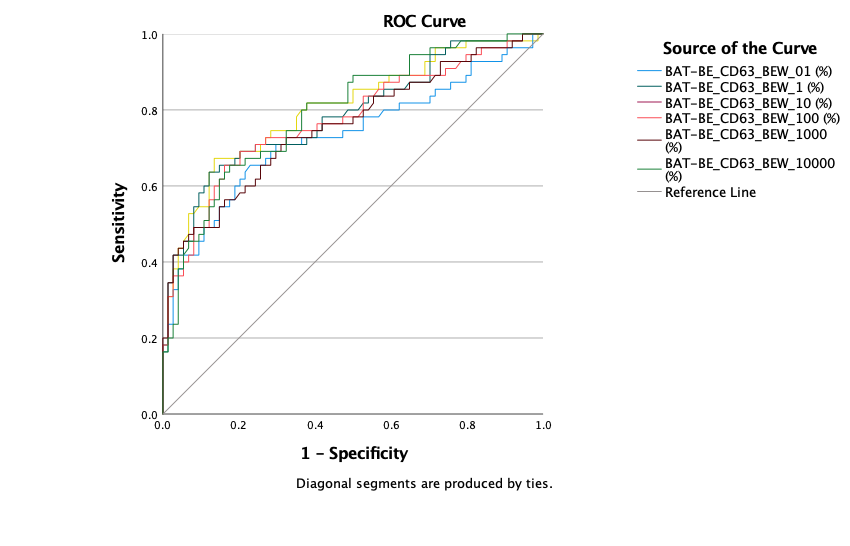


| Test Result Variable(s) | Area | 95% Confidence Interval |  |
| --- | --- | --- | --- |
|  |  | Lower Bound | Upper Bound |
| CD63_BEW_01 (%) | .732 | .640 | .824 |
| CD63_BEW_1 (%) | .787 | .705 | .869 |
| CD63_BEW_10 (%) | .803 | .724 | .882 |
| CD63_BEW_100 (%) | .774 | .690 | .859 |
| CD63_BEW_1000 (%) | .758 | .672 | .844 |
| CD63_BEW_10000 (%) | .794 | .716 | .872 |

1. CD203c and egg white extract


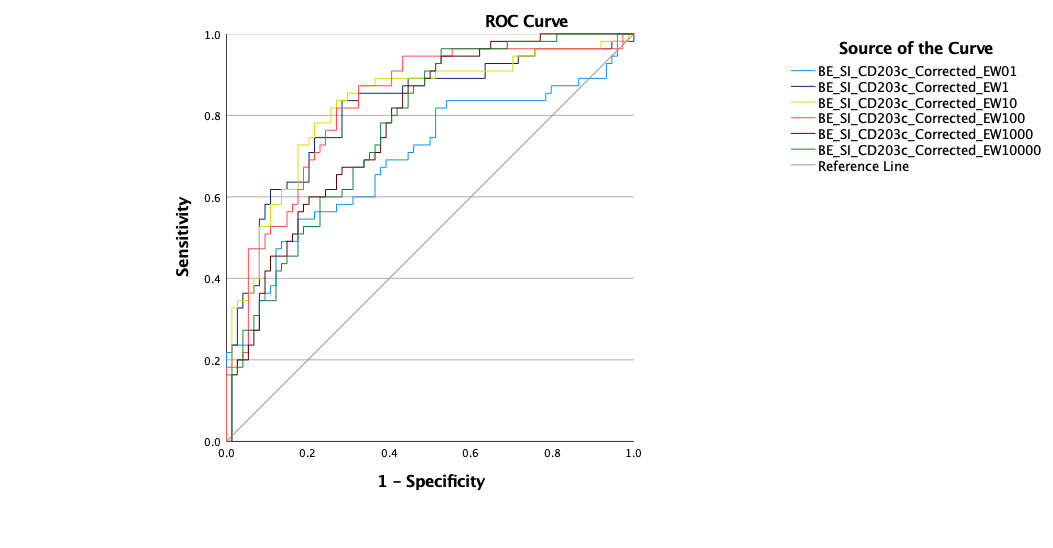


| Test Result Variable(s) | Area | 95% Confidence Interval | |
| --- | --- | --- | --- |
|  |  | Lower Bound | Upper Bound |
| SI_CD203c_Corrected_EW01 | .696 | .600 | .792 |
| SI_CD203c_Corrected_EW1 | .815 | .738 | .892 |
| SI_CD203c_Corrected_EW10 | .827 | .751 | .902 |
| SI_CD203c_Corrected_EW100 | .826 | .752 | .899 |
| SI_CD203c_Corrected_EW1000 | .778 | .700 | .856 |
| SI_CD203c_Corrected_EW10000 | .770 | .690 | .849 |

1. CD203c and baked egg white


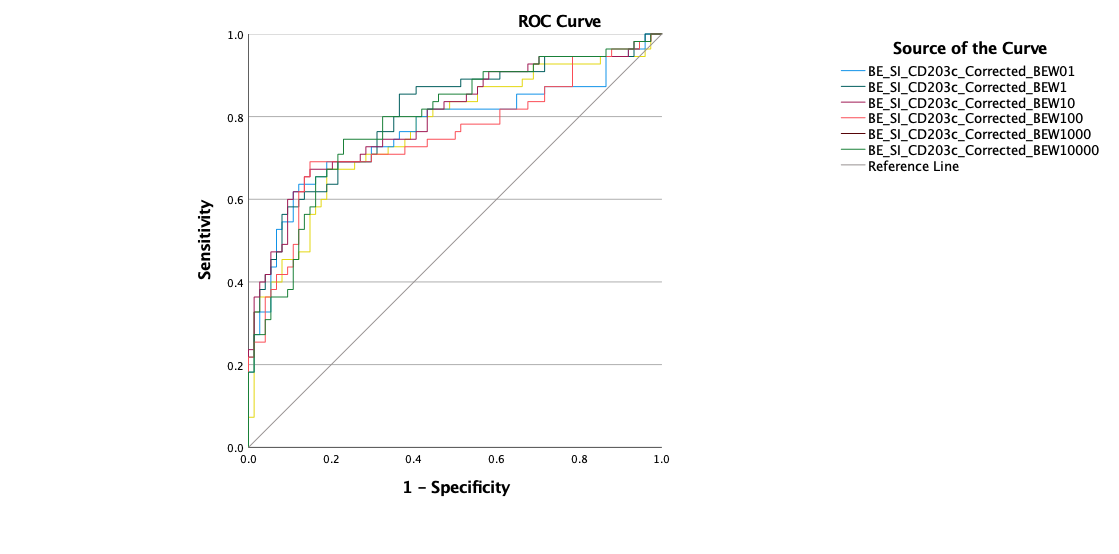


| Test Result Variable(s) | Area | 95% Confidence Interval | |
| --- | --- | --- | --- |
|  |  | Lower Bound | Upper Bound |
| BE_SI_CD203c_Corrected_BEW01 | .766 | .677 | .856 |
| BE_SI_CD203c_Corrected_BEW1 | .803 | .723 | .882 |
| BE_SI_CD203c_Corrected_BEW10 | .796 | .715 | .877 |
| BE_SI_CD203c_Corrected_BEW100 | .751 | .661 | .842 |
| BE_SI_CD203c_Corrected_BEW1000 | .766 | .681 | .852 |
| BE_SI_CD203c_Corrected_BEW10000 | .789 | .709 | .870 |
